# *FGFR3*::*TACC3* fusions in head and neck carcinomas: a study of nine cases highlighting phenotypic heterogeneity, frequent HPV association, and a morphologically distinct subset in favor of a putative entity

**DOI:** 10.1007/s00428-024-03940-3

**Published:** 2024-10-10

**Authors:** Abbas Agaimy, Cristina R. Antonescu, Diana Bell, Gerben E. Breimer, Josephine K. Dermawan, Lennart A. Kester, Jan Laco, Johannes A. Rijken, Rumeal D. Whaley, Robert Stoehr, Thomas Cramer, Justin A. Bishop

**Affiliations:** 1 Institute of Pathology, Erlangen University Hospital, Friedrich Alexander University of Erlangen-Nuremberg, Krankenhausstrasse 8-10, 91054 Erlangen, Germany; 2 Comprehensive Cancer Center, European Metropolitan Area Erlangen-Nuremberg (CCC ER-EMN), Friedrich Alexander University of Erlangen-Nuremberg, Erlangen, Germany; 3 Department of Pathology, Memorial Sloan Kettering Cancer Center, New York, NY USA; 4 Head and Neck/Endocrine Pathology Center of Excellence, Division of Anatomic Pathology, University of Pittsburgh Medical Center, Pittsburgh, PA USA; 5 Department of Pathology, University Medical Center Utrecht, Utrecht, The Netherlands; 6 Department of Pathology and Laboratory Medicine Institute, Cleveland Clinic, Cleveland, OH USA; 7 Department of Head and Neck Surgical Oncology, University Medical Center Utrecht, Utrecht, The Netherlands; 8 The Fingerland Department of Pathology, Charles University Faculty of Medicine in Hradec Kralove and University Hospital Hradec Kralove, Hradec Kralove, Czech Republic; 9 Princess Máxima Center for Pediatric Oncology, Utrecht, The Netherlands; 10 Department of Laboratory Medicine and Pathology, Mayo Clinic, Rochester, MN USA; 11 Department of Otorhinolaryngology, Head and Neck Surgery, Bundeswehrkrankenhaus Berlin, Berlin, Germany; 12 Department of Pathology, University of Texas Southwestern Medical Center, Dallas, TX USA

**Keywords:** Sinonasal, Gene fusions, RNA sequencing, FGFR, Targeted therapy, Tyrosine kinase inhibitor

## Abstract

The *FGFR3*::*TACC3* fusion has been reported in subsets of diverse cancers including urothelial and squamous cell carcinomas (SCC). However, the morphology of *FGFR3*::*TACC3*-positive head and neck carcinomas has not been well studied and it is unclear if this fusion represents a random event, or if it might characterize a morphologically distinct tumor type. We describe nine *FGFR3*::*TACC3* fusion–positive head and neck carcinomas affecting six males and three females aged 38 to 89 years (median, 59). The tumors originated in the sinonasal tract (*n* = 4), parotid gland (*n* = 2), and one case each in the oropharynx, submandibular gland, and larynx. At last follow-up (9–21 months; median, 11), four patients developed local recurrence and/or distant metastases, two died of disease at 11 and 12 months, one died of other cause, one was alive with disease, and two were disease-free. Three of six tumors harbored high risk oncogenic HPV infection (HPV33, HPV18, one unspecified). Histologically, three tumors revealed non-keratinizing transitional cell-like or non-descript morphology with variable mixed inflammatory infiltrate reminiscent of mucoepidermoid or *DEK*::*AFF2* carcinoma (all were HPV-negative), and three were HPV-associated (all sinonasal) with multiphenotypic (1) and non-intestinal adenocarcinoma (2) pattern, respectively. One salivary gland tumor showed poorly cohesive large epithelioid cells with prominent background inflammation and expressed AR and GATA3, in line with a possible salivary duct carcinoma variant. Two tumors were conventional SCC. Targeted RNA sequencing revealed an in-frame *FGFR3*::*TACC3* fusion in all cases. This series highlights heterogeneity of head and neck carcinomas harboring *FGFR3*::*TACC3* fusions, which segregates into three categories: (1) unclassified HPV-negative category, morphologically distinct from SCC and other entities; (2) heterogeneous group of HPV-associated carcinomas; and (3) conventional SCC. A driver role of the *FGFR3*::*TACC3* fusion in the first category (as a potential distinct entity) remains to be further studied. In the light of available *FGFR*-targeting therapies, delineation of these tumors and enhanced recognition is recommended.

## Introduction

The genes encoding the fibroblast growth factor receptors (FGFR1, FGFR2, FGFR3, and FGFR4), mapping to different chromosomes, have been increasingly recognized to be parts of gene fusions involving diverse fusion partners and occurring across a variety of common and rare cancer types in different organs, with generally low but entity-dependent frequencies [1–3]. These fusion genes have received special clinical attention due to their potentially targetable nature [4–7], and because some of them have emerged as resistance mechanisms in patients treated by EGFR-targeting tyrosine kinase inhibitors (TKI) [8, 9]. Among these *FGFR* fusions, those involving *FGFR3* and the transforming acidic coiled-coil containing protein 3 gene (*TACC3*) are generally rare, albeit they represent the most frequently encountered tyrosine kinase fusion [1–3]. They have been detected more frequently in urothelial carcinoma (12%) [10, 11] and in small subsets of pulmonary (0.6% and 3.5% of adenocarcinoma and SCC, respectively) [9, 12, 13], esophageal (1.4–2.1%) [14], cervical (1.3–1.9%) [15, 16], head and neck (HNSCC; 3.7% [17]), and rare nasopharyngeal [17] carcinomas as well glioblastoma (3.1–4.1%) [18], and small subsets of other cancer types [19, 20]. In non-urological cancers, most of *FGFR3*::*TACC3* fusion–positive tumors were reportedly SCC.

However, in contrast to the well-known phenotype-genotype correlations observed in most gene fusion–driven neoplasms, there is still insufficient data on the morphological characteristics of *FGFR3*::*TACC3* fusion–positive head and neck carcinomas and whether they display any degree of phenotype-genotype correlation that might be utilized to identify or suspect potential cases based on initial morphological evaluation in routine practice. Moreover, the possibility that some head and neck carcinomas with this fusion might correspond to a specific entity has not been addressed. We herein describe nine head and neck carcinomas identified in our files carrying the *FGFR3*::*TACC3* fusion in an attempt to characterize their morphological spectrum.

## Material and methods

Three cases were initially screened for possible gene fusions by one of the authors (A.A.) during consultation based on their unclassified or confusing morphology, which was suggestive of fusion-associated carcinomas (*MAML2* or *DEK*::*AFF2* fusion) but did not fit into a well-defined tumor category. Additional cases were then searched in the molecular data base of our institutions. None of the cases has been reported before.

### Next-generation sequencing

In cases 1 to 3 and case 8, RNA was isolated from formalin-fixed paraffin-embedded (FFPE) tissue sections using RNeasy FFPE Kit of Qiagen (Hilden, Germany) and quantified spectrophotometrically using NanoDrop-1000 (Waltham, USA). Molecular analysis was performed using the TruSight RNA Fusion panel (Illumina, Inc., San Diego, CA, USA) with 500 ng RNA as input according to the manufacturer’s protocol. Libraries were sequenced on a MiSeq (Illumina, Inc., San Diego, CA, USA) with > 3 million reads per case, and sequences were analyzed using the RNA-Seq Alignment workflow, version 2.0.1 (Illumina, Inc., San Diego, CA, USA). The Integrative Genomics Viewer (IGV), version 2.2.13 (Broad Institute), was used for data visualization [21]. In case 4, total RNA extracted by RNeasy Plus Mini (Qiagen) from fresh frozen tumor tissues was subjected to whole transcriptome analysis as described previously [22]. In case 5, targeted next-generation sequencing (NGS) consisted of DNA sequencing of 523 genes to detect single-nucleotide variants (SNVs), copy number variations (CNVs), microsatellite instability (MSI), and tumor mutation burden (TMB), and RNA sequencing of 165 genes to detect rearrangements of known and novel gene partners as described recently [23]. Case 6 was tested with a custom amplicon-based NGS assay using the Archer FusionPlex (Archer, Boulder, CO) standard protocol [24]. For case 7, RNA was isolated from FFPE tissue sections, and RNA-Seq data was obtained using the methods described by Hehir-Kwa et al. [25]. Case 9 was tested using the Illumina TruSight Oncology 500 [TSO500] panel (MayoComplete Solid Tumor Panel), as previously described [26], which was performed to evaluate for therapeutically relevant alterations including microsatellite instability. This assay includes a DNA subpanel for the detection of sequence alterations in 515 genes and amplification of 59 genes as well as an RNA subpanel for the detection of fusions involving 55 genes.

### HPV testing

The HPV status and subtyping have been assessed using either in situ hybridization methods [27] or via a DNA-based method [28, 29] as described previously. Case 3 was tested using a AmoyDx® Human Papillomavirus (HPV) Genotyping Detection Kit which is a real-time PCR assay enabling qualitative detection of up to 19 high-risk HPV DNA (HPV 16, 18, 26, 31, 33, 35, 39, 45, 51, 52, 53, 56, 58, 59, 66, 68, 70, 73, and 82).

## Results

### Clinical and demographic features

The main clinicopathological features are summarized in Table 1. The affected patients were six males and three females aged 38 to 89 years (median, 59). The tumors originated in the nasal cavity/sinuses (*n* = 4), the parotid gland (*n* = 2), and one case each in the oropharynx, submandibular gland, and larynx. Most tumors presented as large masses measuring 1 to 8 cm in size (median, 4.8). Radical surgical resection was the initial treatment in eight patients; details on adjuvant therapy were not available. One recent case was maxillary sinus tumor which was treated with pembrolizumab in palliative intention. Follow-up was available for six patients (range, 9–21 months; median, 11). Four patients developed local recurrence and/or distant metastases; two of them died of disease at 11 and 12 months, one was alive with metastatic disease at 18 months, and one remained disease-free at 21 months following re-excision of local recurrence. One patient was disease-free at 9 months. One patient died of an unrelated cause (stroke) at 10 months.

**Table 1:** *FGFR3::TACC3* fusion–positive head and neck carcinomas (*n* = 9)

No	Age/sex	Site/size in cm	Original/submitted diagnoses	Histological pattern	p16	HPV	Treatment	Outcome	Fusion
1	53/M	Nasal cavity/3.6	Mucoepidermoid vs DEK::AFF2 carcinoma	TCC-like with mucinous features	neg	neg	Surgery; surgery + adjuvant radiotherapy for recurrent	Local recurrence (11 mo), NED (21 mo)	*FGFR3::TACC3*
2	78/F	Larynx/ < 1	Inverted papilloma vs carcinoma NOS?	TCC-like SCC	neg	neg	Surgery	NA	*FGFR3::TACC3*
3	76/F	Parotid/8	DEK::AFF2 carcinoma?	DEK::AFF2 carcinoma-like	neg	neg	Surgery	LR + wide-spread MTS, DOD (11 mo)	*FGFR3::TACC3*
4	58/M	Parotid/7.6	k-SCC	Focally keratinizing SCC	NA	NA	Surgery	DOD (12 mo)	*FGFR3::TACC3*
5	59/M	SMG/4.8 cm	Salivary duct carcinoma ex PA, GATA3 + ; Her2: 2 +	Diffuse pattern with prominent inflammation, focal PA-like sclerosis	neg	neg	Surgery, T3N2cM1	AWD 16 months (local rec, liver, lung)	*FGFR3::TACC3* *FGFR3::ITPR1*
6	50/M	Sinonasal/NA	Multiphenotypic HPV + carcinoma	AdCC-like + TCC-like	pos	HPV33	Surgery + CRT	NED (9 mo)	*FGFR3::TACC3*
7	77/M	Oropharynx/NA	k-SCC	Keratinizing SCC	neg	NA	T2N3bM1; systemic therapy—pembrolizumab/carboplatin/capecitabine (palliative)	DUD (10 mo) Stroke	*FGFR3::TACC3*
8	38/F	Sinonasal/NA	High-grade non-ITAC	Non-ITAC + solid areas	neg	HPV18	Surgery	Recent case	*FGFR3::TACC3*
9	89/M	Sinonasal/NA	Mucoepidermoid carcinoma	Unclassified mucinous adenocarcinoma	pos	Pos^a^	Pembrolizumab (palliative)	Recent case	*FGFR3::TACC3*

*AdCC* adenoid cystic carcinoma; *AWD* alive with disease; *CRT* chemoradiotherapy; *DOD* died of disease; *DUD* died of unrelated disease; *k-SCC* keratinizing squamous cell carcinoma; *ITAC* intestinal-type adenocarcinoma; *LR* local recurrence; *NA* not available; *NED* no evidence of disease; *PA* pleomorphic adenoma; *SMG* submandibular gland; *TCC* transitional cell carcinoma

^a^Exact high-risk HPV subtype not determined by the assay

### Pathological findings

The initial diagnoses submitted by the primary pathologists or considered by the referral pathology were non-keratinizing transitional cell-like carcinoma (suggestive of *DEK*::*AFF2* carcinomas) in two cases and mucoepidermoid carcinoma in two cases. One laryngeal tumor was considered an inverted papillary epithelial tumor/papilloma by the primary pathologist and as a transitional cell-like carcinoma with prominent inflammation, suggesting variant mucoepidermoid carcinoma after referral pathology. Two tumors were initially diagnosed as keratinizing SCC, and one case each as salivary duct carcinoma ex pleomorphic adenoma, HPV-related multiphenotypic sinonasal carcinoma, and high-grade non-intestinal adenocarcinoma with solid component.

**Fig. 1:**
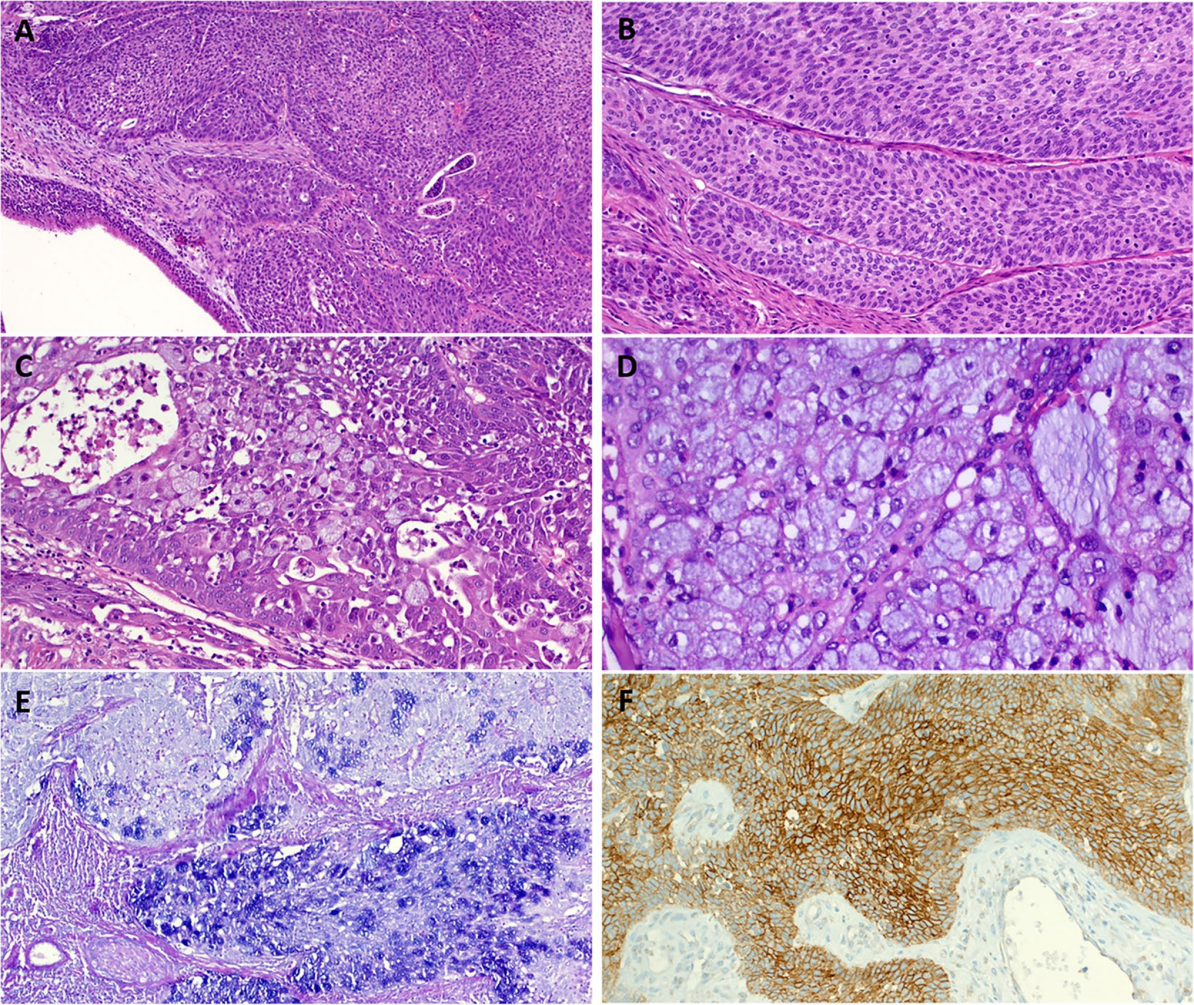
Representative example of the morphological findings in *FGFR3*::*TACC3* fusion unclassified head and neck carcinomas (case 1). (**A, B**) This sinonasal tumor showed invasive growth of monomorphic epidermoid or squamoid cells disposed into macrotrabeculae and solid nests (**B**). A minor mucinous cell component was seen in the primary tumor (**C**) and was more striking in the recurrent tumor (**D**), highlighted by Alcian–PAS stain in **E**. **F** Strong membranous reactivity with FGFR3

**Fig. 2:**
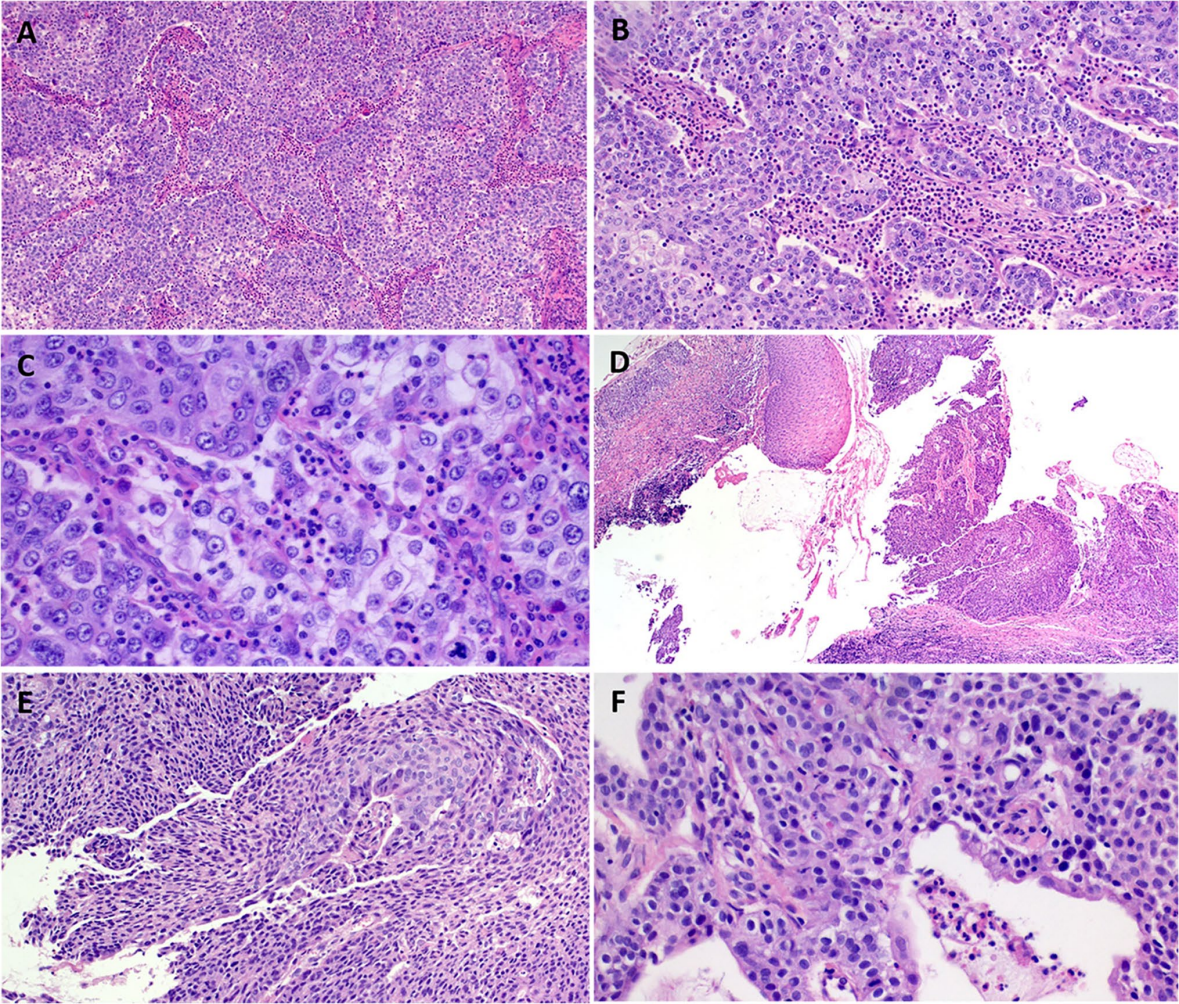
Another example of *FGFR3*::*TACC3* fusion unclassified head and neck carcinoma (case 3). This parotid tumor was composed of monomorphic epidermoid cells arranged into irregular branching aggregates (**A**). Transitional-like cell morphology and prominent neutrophilic inflammation mimicking *DEK*::*AFF2* carcinoma is noted in (**B**). (**C**) Higher magnification showing focal cytoplasmic clearance. (**D**) This laryngeal lesion showed invasive as well as superficial CIS-like growth with prominent peritumoral inflammation. (**E**) Higher magnification showing transitional cell morphology, note focal elongated pseudoglandular cells in (**E**) and papillary growth in** (F**)

Histologically, the tumors segregated into three main categories. The first group (seen in three cases) did not conform to any defined carcinoma type. These tumors showed diffuse sheets and confluent nests of medium-sized non-descript basophilic to pale-eosinophilic epithelial cells showing variable resemblance to squamous epithelium but lacking distinct squamous features (intercellular bridges, prominent cytoplasmic eosinophilia, keratinization) and associated with prominent (predominantly mononuclear in 2 and neutrophilic in 1) mixed inflammatory reaction in the background stroma and at the periphery of the neoplasm, reminiscent of sinonasal *DEK*::*AFF2* carcinomas and of monomorphic (variant) mucoepidermoid carcinoma (Figs. 1 and 2A, B, C). The recurrent tumor of case 1 showed prominent cytoplasmic mucinous material that was Alcian-PAS-positive, closely mimicking mucin cell-rich mucoepidermoid carcinoma (Fig. 2D, E). This mucinous component was only focally present in the primary tumor (Fig. 2C). The laryngeal tumor showed transitional cell-like syncytial growth mimicking inverted/endophytic solid-papilloma pattern with prominent lymphoid reaction mainly at the tumor-stromal interphase (Fig. 2D, E, F). This case was judged by the primary pathologist as suggestive of an inverted papilloma-like lesion and as a potential variant mucoepidermoid carcinoma after second opinion review, justifying molecular testing. All three tumors in this category lacked high-risk HPV by molecular methods.

**Fig. 3:**
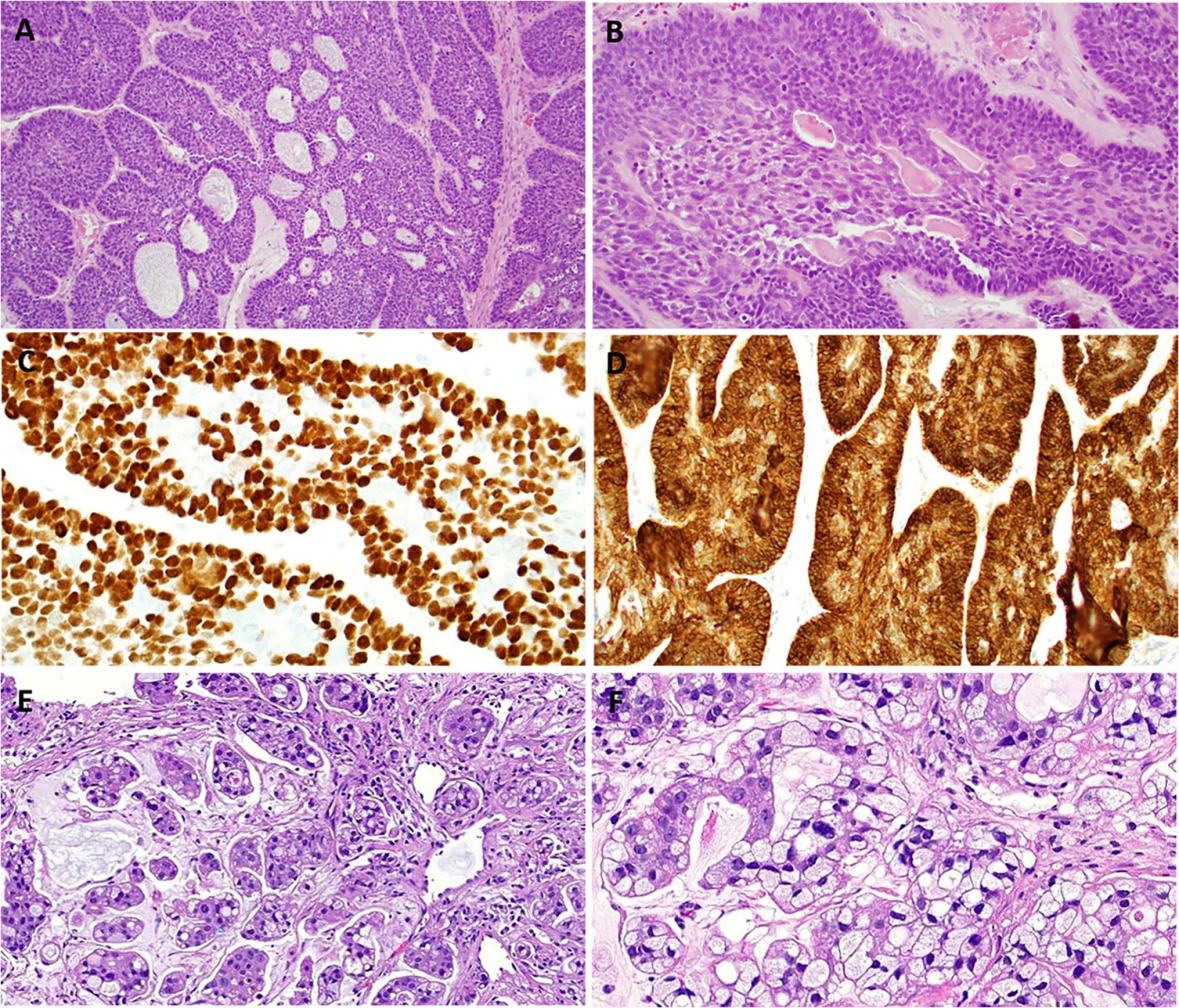
Representative examples of HPV + head and neck carcinomas with *FGFR3*::*TACC3* fusions. **A** This sinonasal multiphenotypic carcinoma (case 6) showed adenoid cystic-like features with variable focal transitional-like morphology **B**. **C** The tumor cells were highly positive for high-risk HPV 33 (ISH). **D** Strong membranous reactivity with FGFR3 (Case 6). **E** and **F** HPV-positive unclassified sinonasal adenocarcinoma with mucinous features (Case 9)

In the second category (including three sinonasal tumors), one tumor revealed adenoid cystic-like morphology with variable transitional-like epithelial features, altogether consistent with multiphenotypic sinonasal carcinoma (Fig. 3A-D). This tumor was also positive for HPV33 (Fig. 3C). The second tumor showed non-intestinal glandular growth with high-grade features and an abrupt transition to a solid undifferentiated looking tumor component. This case was also positive for oncogenic HPV (HPV18). The third case showed features of mucinous adenocarcinoma (Fig. 3E, F) and harbored a high-risk HPV (exact subtype not determined).

**Fig. 4:**
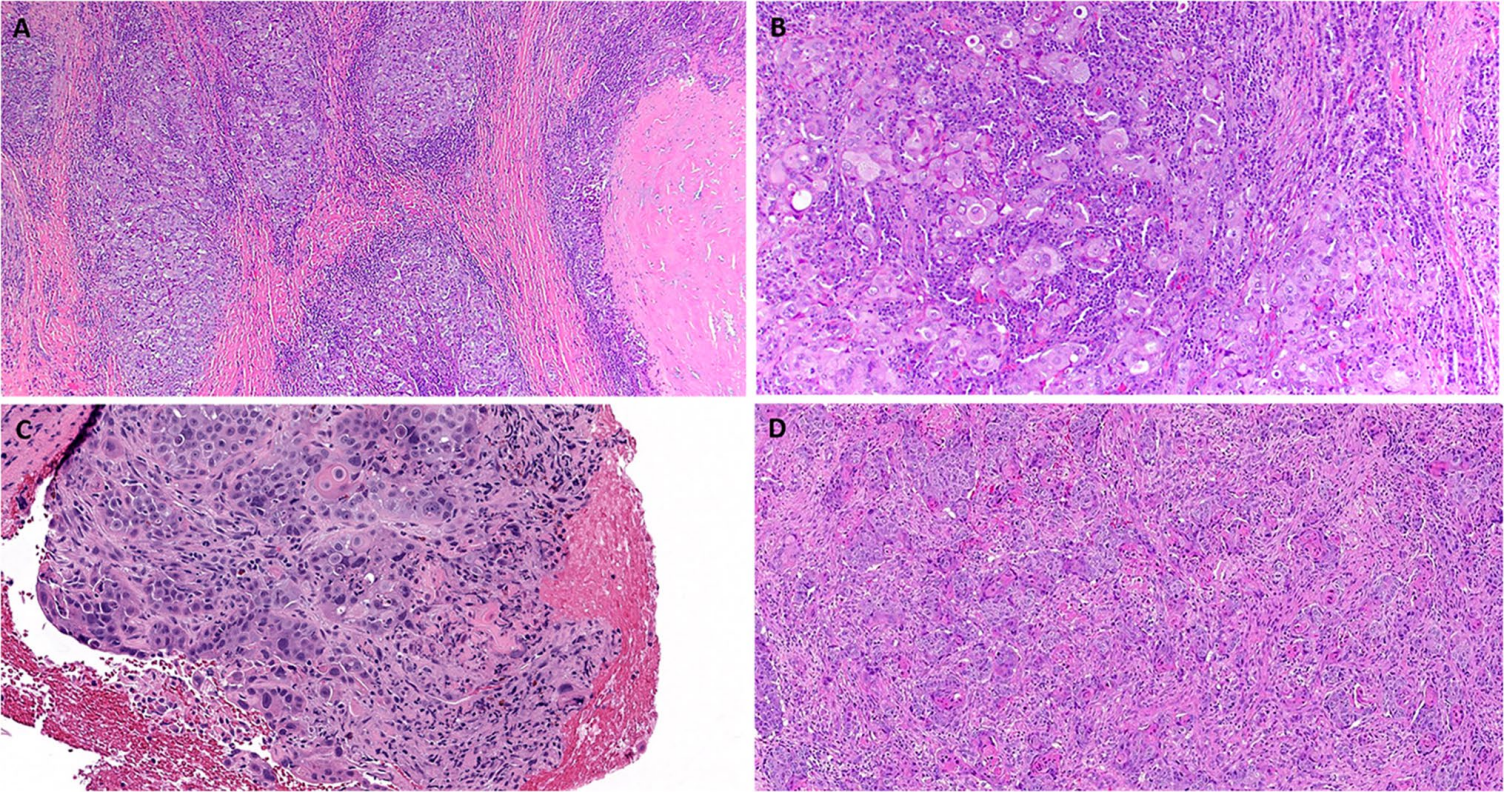
Representative examples of the heterogeneous morphological findings in *FGFR3*::*TACC3* fusion head and neck carcinomas. **A** This submandibular gland tumor (Case 5) showed focal dense sclerosis surrounded by large epithelioid cells arranged into loose aggregates mimicking carcinoma ex pleomorphic adenoma, note prominent mononuclear inflammation. **B** Higher power showed large non-descript epithelioid cells within prominent inflammatory background; no features of classical SDC were seen. **C** (case 4) and **D** (case 7) Conventional squamous cell carcinoma with variable subtle to prominent keratinization

The pattern seen in one submandibular gland tumor was obviously overlapping with the above three tumors, but the neoplastic cells were larger and epithelioid, associated with prominent mononuclear background inflammation (Fig. 4A, B). The central part of this tumor showed a nodular sclerotic hyalinized zone that was initially interpreted as possible preexistent pleomorphic adenoma, but unequivocal viable adenoma tissue was not detected. This tumor showed, in addition, overlap with poorly cohesive salivary duct carcinoma, a possibility further enhanced by reactivity for AR and GATA3, but not p40.

The last subgroup was represented by two conventional SCC with variable keratinization that originated in the parotid gland and the oropharynx (Fig. 4C, D). Notably, all patients did not have evidence of other, histologically similar primary tumors in other locations. In particular, patients with parotid gland tumors had no evidence of other primary carcinoma in the head and neck skin or any other site.

### Immunohistochemical findings

Different immunostains were performed on a case-to-case basis due to the consultation nature of most cases and the respective differential diagnoses raised. All tumors (except case 5) showed a squamous immunophenotype with diffuse expression of pankeratin, CK5/6, and p63/p40 (not shown). Case 5 expressed the androgen receptor and GATA3 but was negative for p40. Case 9 (unclassified sinonasal mucinous adenocarcinoma) expressed CK7 and NKX 3.1 and was negative for AR, p40, TTF1, CDX2, CK20, GATA3, uroplakin II, and HER2. Cases 1 and 6 were tested for FGFR3 immunohistochemistry and showed strong and diffuse membranous staining (Figs. 1F and 3D).

### Molecular results

HPV testing was performed successfully on seven tumors; three of them were positive for high-risk HPV. HPV33 was detected in one multiphenotypic sinonasal carcinoma and HPV18 in the sinonasal tumor with admixture of high-grade non-intestinal adenocarcinoma and solid pattern carcinoma. The third case was unclassified mucinous sinonasal adenocarcinoma. p16 was block-type positive in two of the three HPV-positive cases (see below) and negative in six cases; all of them, but one case, were HPV-negative and two with unknown HPV status. Notably, all three tumors that expressed squamous cell markers but were otherwise unclassified (cases 1, 2, and 3) tested negative for HPV.

Targeted RNA sequencing revealed an *FGFR3*::*TACC3* gene fusion in all cases. However, the breakpoints for both genes were heterogeneous among the cases (Table 2). Case 5 harbored an additional *FGFR3::ITPR1* fusion. DNA testing of this case revealed a *TP53* mutation (c.626_627del, p.R209Kfs*6). An additional fusion *RPS6KB1*(exon 4)::*VMP1*(exon 11) was detected in case 9. Additional alterations in this recent case included amplification of 12q13-15 (CDK4, GLI1, and MDM2) and AURKA amplification.

**Table 2:** Gene breakpoints and exons involved in *FGFR3::TACC3*-rearranged head and neck carcinomas

**No**	*FGFR3* breakpoints	*FGFR3* breakpoints
1	*FGFR3* Exon 15	*TACC3* Exon 14
2	*FGFR3* Exon 17	*TACC3* Exon 11
3	*FGFR3* Exon 17	*TACC3* Exon 6
4	*FGFR3* Exon 17	*TACC3* Exon 10
5	*FGFR3* Exon 17	*TACC3* Exon 10
6	*FGFR3* Exon 17	*TACC3* Exon 11
7	*FGFR3* Exon 18	*TACC3* Exon 4
8	*FGFR3* Exon 17	*TACC3* Exon 14
9	*FGFR3* Exon 17	*TACC3* Exon 11

## Discussion

Gene fusions involving the fibroblastic growth factor receptors (FGFRs) 1–4 occur in three main oncogenetic settings: (1) as a sole driver, mutually exclusive with other known drivers, in certain tumor entities; (2) as oncogenically active additional drivers concurrent with other genetic events as in some HPV-associated cervical and other virally induced cancers; and (3) as a mechanism of secondary resistance in tyrosine kinase inhibitor (TKI)-pretreated carcinomas [1–3, 8]. Recently, *FGFR::TACC* fusions have been reported in a variety of non-epithelial cancers, including *FGFR1::TACC1* fusions as a novel alternative genetic driver in rhabdomyosarcoma [30], and *FGFR2::TACC2* fusions as a mechanism of multidrug resistance in TKI-treated gastrointestinal stromal tumors (GIST) [31].

Beside their role as resistance mechanisms, *FGFR* fusions have received special clinical attention due to their potentially targetable nature using diverse available anti-FGFR TKIs [4–7]. Although generally uncommon, fusions involving *FGFR3* and the transforming acidic coiled-coil containing protein 3 gene (*TACC3*) represent one of the most frequently encountered tyrosine kinase fusions [1–3]. Besides glioblastoma [1, 18], they have been detected in small subsets of carcinomas of urothelial, head and neck, pulmonary, esophageal, cervical, and other organ origin [9–17, 19, 20].

Although the striking variation in the frequency of the *FGFR3*::*TACC3* fusions among different cancer types in different organs is suggestive of a potential morphomolecular correlation, to date no studies are available that critically evaluate the morphological features of *FGFR3*::*TACC3*-altered carcinomas. However, several studies have pointed out that most of *FGFR3*::*TACC3* fusion–positive tumors represent either SCC or urothelial carcinomas with squamous-like phenotype [10, 11]. Moreover, an *FGFR3*::*TACC3* fusion has been recently reported in one of 11 malignant Brenner tumors of the ovary, an entity known to show transitional cell-like morphology and squamous immunophenotype [32]. Notably, emergence of the *FGFR3*::*TACC3* fusion as a resistance mechanism in TKI-treated EGFR-mutated lung adenocarcinoma was associated with a tendency towards SCC-like transdifferentiation [9].

Taken together, it is remarkable that most *FGFR3*::*TACC3* fusion carcinomas display either a transitional cell-like morphology (including urothelial carcinoma), a squamous cell phenotype (SCC), or combined features of both [10, 11]. This is also true for our current series where most of the tumors represented either unclassified carcinomas with transitional cell-like morphology and squamous cell phenotype or bona fide SCC. Furthermore, some studies have suggested distinct clinicopathological characteristics of *FGFR3*-fused NSCLC including more frequent association with smoking, significantly larger tumor size, and more frequent poor differentiation than those without *FGFR* fusions [13].

In the head and neck, only limited studies are available on the prevalence and clinicopathological characteristics of carcinomas harboring *FGFR3*::*TACC3* fusions. Overall, this fusion was detected in a total of 22 head and neck carcinomas reported in several studies [33]: 2 cases in the survey study by Wu et al. [2], 2 of 411 HNSCC in the TCGA dataset [34], 1 of 47 HPV-positive oropharyngeal SCC [35], 1 (nasal) of 24 poorly differentiated head and neck neuroendocrine carcinomas [36], 4 of 159 nasopharyngeal carcinomas (2.5%), and 7 of 308 HNSCC (3.7%) analyzed by Yuan et al. [17]. Two additional cases have been identified via sequencing of 279 (172 oral, 33 oropharyngeal, and 72 laryngeal) HNSCC samples from the TGCA dataset; both tumors were HPV-positive [37]. An additional nasopharyngeal tumor lacking EBV was reported more recently [23]. During the preparation of the current study, Chu et al. published two head and neck carcinomas harboring *FGFR*::*TACC* fusions: an HPV-related multiphenotypic sinonasal carcinoma carrying an *FGFR2*::*TACC2* fusion with concurrent non-16/18 high-risk HPV, and one *FGFR3*::*TACC3* fusion–positive keratinizing SCC of the parotid gland [38].

Except for the nasopharyngeal tumors and the multiphenotypic sinonasal carcinomas, the vast majority of *FGFR3::TACC3*-positive reported cases were supposed to have represented SCC. However, no detailed morphological features of these carcinomas carrying the fusion are available, and it is not clear if the SCC diagnosis was based on morphological or immunophenotypic features. Moreover, the majority of these prior studies have performed blind screening of SCC cohorts, which may explain the discrepancy to our current study, where only two of our nine tumors (both conventional SCC) were screened blindly for gene fusions, while the remaining seven tumors were tested by NGS due to their unclassified nature/morphology in the context of primary diagnostic workup.

Our current series is the first one devoted to morphological characterization of head and neck carcinoma harboring the *FGFR3*::*TACC3* fusion. Although limited by the low case number and morphological and anatomic heterogeneity, our study indicates that *FGFR3*::*TACC3* fusion tumors segregate into three major categories. One category represents conventional SCC (two cases), in which the fusion likely represents a random event. We did not observe any morphological hints to the underlying gene fusion in these two cases and the tumors were subjected to targeted RNA sequencing in the context of oncological therapy or blind screening.

The second category is represented by three HPV-associated sinonasal carcinomas. In this category, a heterogeneous morphology was observed including HPV33-associated multiphenotypic sinonasal carcinoma, one sinonasal carcinoma combining high-grade unclassified non-intestinal morphology with solid undifferentiated growth pattern (positive for HPV18) and one sinonasal mucinous adenocarcinoma with undetermined high-risk HPV. Multiphenotypic sinonasal carcinoma is a recently characterized morphologically diverse entity associated with mostly non-16/18 HPV high-risk HPV infection and it combines multiple mostly salivary-like growth patterns [39]. Data on gene fusions in multiphenotypic sinonasal carcinoma is sparse [35]. Recent studies pointed to the role of gene fusions as transforming event in HPV-related carcinogenesis. In particular, activation of the FGFR pathway has been postulated to play a central role in HPV-associated cervical oncogenesis [15, 16]. Moreover, recurrent *UBR5*::*ZNF423* and *FGFR3*::*TACC3* fusions have been detected in 8.3% and 2.5% of nasopharyngeal carcinomas, another rare virally induced head and neck carcinoma type [18, 40]. Another study detected the fusion in one of 47 HPV + oropharyngeal SCC [35]. These observations are in line with a central oncogenic role of the fusion in these virally driven carcinomas and argue against the fusion being merely a passenger alteration.

The last category in our series (three cases) merits special attention. The three tumors could not be classified initially by histology alone, even after seeking second opinion. The targeted RNA sequencing of these three cases was ordered for diagnostic purposes based on their resemblance to other fusion carcinoma (mimicking *DEK*::*AFF3* carcinoma in two cases and mucoepidermoid carcinoma in one). These tumors shared with *DEK*::*AFF2* sinonasal carcinoma the transitional cell-like morphology, the uniform cytology, the squamous immunophenotype, and the presence of prominent intratumoral inflammation [41]. However, some differences exist between the two tumor types, mainly the predominance of mononuclear cells in two of our current cases as opposed to the prominent neutrophilic inflammation (seen in one of three cases) and the more diffuse growth compared to the prominent papillary/papilloma-like growth of the *DEK*::*AFF2* carcinomas, respectively [41, 42]. These three tumors are suggestive of a potential tumor entity driven by the *FGFR3::TACC3* fusion and lacking HPV infection, which merits further delineation in the future when more cases are reported. The striking mononuclear inflammatory reaction in the stromal background and within the tumor cell aggregates in these three tumors might be explained by recent studies which illustrated activation of an inflammatory response pathway resulting from activated FGFR3-MAPK signaling in *FGFR3*::*TACC3* fusion neoplasms [16].

One of our salivary cases with this fusion was originally diagnosed as SDC ex pleomorphic adenoma (PA) based on the variable expression of androgen receptor and presence of a nodular densely sclerosed central zone. However, a genuine PA could not be verified histologically, and a PA-compatible gene fusion was lacking in this case and the tumor morphology was rather nondescript, with diffuse less cohesive growth of medium-sized epithelioid cells associated with prominent diffuse mononuclear inflammation. The *FGFR3*::*TACC3* fusion has been reported in one of 27 metastatic salivary gland carcinomas subjected to next-generation sequencing in one study [5]. That fusion-positive case, which has been reported as SDC, harbored additional driver alterations (*BRCA2* and *PTEN*) in addition to the *FGFR3*::*TACC3* fusion; the morphology of the tumor has not been illustrated [5].

Reviewing previous studies, there seems no differences between the breakpoint variants across different *FGFR3*::*TACC3* fusion cancers [2, 3, 10, 11]. Most tumors including head and neck cancers and our current cases showed in-frame fusions mostly involving exon 18 (less frequently exon 19) of *FGFR3*, fused to exon 6, 8, 10, 11, and 14 and intron10/exon 11 of *TACC3* [2, 3, 10, 11]. All *FGFR3* fusions were in-frame and have involved the whole kinase domain [2–13]. The result of the *FGFR3*::*TACC3* fusion was truncation of the C-terminal region of FGFR3 while the kinase domain was retained and fused to TACC3. Notably, the TACC3 retains its coiled-coil dimerization domain. Being involved in the cross-linkage of the mitotic spindle microtubules to kinetochores, the TACC proteins are essential for mitotic spindle stability and hence for proper cell division [10]. Abnormalities of the TACC3 protein resulting from rearrangements have been shown to result into aneuploidy, chromosomal segregation errors, and cancer progression [10, 43].

The FGFR fusions represent a druggable therapeutic target, irrespective of the presence or absence of concurrent driver events in the tumor [4–7, 16]. Moreover, the presence of the fusion has been shown to herald resistance to anti-EGFR drugs, not only in NSCLC, but also in head and neck cancer [8, 9, 44].

Anti-FGFR3 immunohistochemical antibodies have proven promising in identifying underlying *FGFR3* fusions and might be exploited as initial screening tool in routine. Using FGFR3 immunohistochemistry, Granberg et al. detected *FGFR3* fusions in 10 of 15 strongly stained gliomas, but in none of 36 negatively to moderately positive tumors [45]. These results indicate that not the mere expression but the pattern of expression (strong and diffuse) is likely powerful in detecting the underlying fusion. However, controlled studies on the sensitivity and specificity of the FGFR3 immunohistochemistry in head and neck cancer are not available.

In summary, we described a series of nine head and neck carcinomas carrying an *FGFR3*::*TACC3* fusion. Our results highlight significant heterogeneity of these tumors but point to the existence of three groups of *FGFR3*::*TACC3-*fused head and neck carcinomas: (1) unclassified carcinomas sharing distinctive morphological features in favor of a novel tumor type driven by the fusion, (2) HPV-associated (mostly sinonasal) carcinomas with concurrent *FGFR3::TACC3* fusions, and (3) conventional SCC where the fusion probably represents a random event. Recognition of more cases is mandatory to verify the existence of a distinctive *FGFR3*::*TACC3*-driven carcinoma type and to address the role of targeted therapy in these poorly characterized tumors.

## Data Availability

The datasets generated during and/or analyzed during the current study are not publicly available, but are available from the corresponding author on reasonable request.
